# Attachment to robots and therapeutic efficiency in mental health

**DOI:** 10.3389/fpsyg.2024.1347177

**Published:** 2024-01-31

**Authors:** Mate Szondy, Peter Fazekas

**Affiliations:** ^1^Department of Personality and Clinical Psychology, Pázmány Péter Catholic University, Budapest, Hungary; ^2^Research Unit for Robophilosophy and Integrative Social Robotics, Jewish Charity Hospital, Budapest, Hungary; ^3^Aarhus University, Aarhus, Denmark

**Keywords:** mental health, social robots, treatment gap, attachment formation, levels of attachment

## Abstract

Previous studies in the mental health context have demonstrated that interactions with social robots can improve the mood and cognitive capacities of their users, and enhance their quality of life. In this Perspective article, our goal is to systematize the possible roles of social robots and to point out that different roles require different levels of attachment. We argue that the attachment between the client and the (robot) therapist is a fundamental ingredient of any helping relationship and that the full potential of using social robots in mental health settings can only be realized if the strength of attachment is appropriately correlated with the type of relationship established.

## Introduction: robots in mental health

Social robots are robots that are “designed to interact with people in human-centric terms and to operate in human environments alongside people” (Breazeal et al., 2016). In their interactions with humans, these systems follow the behavioral norms and expectations that are defining features of social interactions, such as emotional expressiveness, verbal communication, user engagement, and an appealing physical appearance (Scassellati et al., 2012).

The rolesocial robots can play in delivering mental health care interventions for children and older adults (especially those with ASD and dementia, respectively) has been widely studied (for recent reviews see Cifuentes et al., 2020, Marchetti et al., 2022). These investigations have shown that social robots can be effective in engaging users, improving their mental health, mood and cognitive capacities, and enhancing their quality of life.

According to a recent review, in mental health settings, social robots are typically used in three major contexts: acting as therapists/coaches, mediators, or assistants (David et al., 2014). In this Perspectives article, our goal is to refine this distinction about the possible roles of social robots and to point out that different roles require different levels of attachment. We will argue that the attachment between the client and the robot is a fundamental ingredient of any helping relationship and that the full potential of using social robots in mental health settings can only be realized if the strength of attachment is appropriately correlated with the type of relationship established.

## The role of attachment in human-led therapy

Thinking about attachment in robot therapy should be guided by our understanding of the role attachment plays in traditional (that is, human-led) psychotherapy.

An influential framework from this perspective is Bowlby’s attachment theory (Bowlby, 1982). This theory posits that the psychotherapeutic relationship can offer a significant tool that aids the client in transitioning from insecure to secure attachment. Bowlby suggests that the role of the therapist is to act as an attachment figure by creating a secure base to enable the exploration of attachment-related experiences and to provide corrective emotional experiences to disconfirm insecure working models (Sherman et al., 2015; Degnan et al., 2016; Fraley and Roisman, 2019).

More recent research indicates that clients can develop more secure attachments as a result of therapy (Taylor et al., 2015; Gagliardi, 2022). From this perspective, the central question is whether the therapeutic relationship can truly function as an attachment relationship. Mallinckrodt (2010) identifies five salient characteristics of attachment relationships and analyses evidence to ascertain if the therapeutic relationship meets these criteria. The five key characteristics are: (1) the attachment figure is a *target for proximity seeking*; (2) the attachment figure supplies a *safe haven* to offer comfort during periods of distress; (3) the attachment figure lends a *sense of security* that allows the individual to explore; (4) the individual experiences *separation anxiety* when the attachment figure is unavailable; and (5) the attachment figure is *stronger and wiser* than the individual. Although the final aspect is not essential for adult attachment relationships, the rest are observable in therapeutic relationships (Mallinckrodt, 2010). That is, therapeutic relationships do have the potential to serve as attachment relationships, hence they hold the capacity to modify insecure internal working models.

Although some therapeutic relationships manifest all the critical features of attachment, this does not imply that most therapeutic relationships are attachment relationships. The efficacy of many therapeutic approaches does not require attachment, and time constraints on therapeutic relationships often prevent the formation of a fully secure attachment. Still, improving the client’s attachment style via the development of proximity seeking, safe haven, and secure base may be critical goals for the therapist (Mallinckrodt, 2010).

Offering a different perspective, Saunders et al. (2011) propose that the therapist serves as an “alternative support figure” (i.e., adults apart from parents who provide support). If the patient manages to foster a trusting relationship with the therapist, the patient can nurture feelings of worthiness, cultivate a more positive self-perception, and potentially develop a stronger capacity for reflective functioning. Reflective functioning is defined as one’s ability to contemplate their own experiences to draw conclusions about their mental state and that of others (Fonagy et al., 1996).

## Attachment to robots and therapy

Objects can elicit powerful emotions that extend beyond liking (Norman, 2004). People can become attached to objects, such that they feel a psychological or emotional bond with them (Norberg and Rucker, 2021). Neuroimaging data show that mental processes similar to those involved in perceiving humans are triggered when people anthropomorphise non-human objects (Waytz et al., 2010). Attributing humanlike properties and characteristics to nonhuman agents and objects is at the core of anthropomorphism (Epley et al., 2007). Anthropomorphism can in turn transform human-to-object interactions into human-to-human-like interactions and result in object attachment by fulfilling human needs related to comfort and pleasantness, self-identity, and self-efficacy (Wan and Chen, 2021). Such formation of psychological and emotional bonds (i.e., attachment) can even take the form of companionship (perceived) friendship, or love (e.g., Ki et al., 2020).

Findings reporting empathy with robot ‘pain’ (Suzuki et al., 2015), concerns and pity for robots that are tortured (Rosenthal-von der Pütten et al., 2013), hesitation to strike a robot (Darling et al., 2015) and an overlap in neural activity associated with empathy toward humans and empathy toward robots (Rosenthal-von der Pütten et al., 2013; Chin et al., 2023) indicate that humans do feel empathy for robots. It has also been shown that inducing empathy triggers prosocial human behavior (i.e., increased helpfulness) toward a robot (Kühnlenz et al., 2013), that people seem to be inclined to help a robot find its way (Weiss et al., 2010), and that they empathize with a robot when something bad happens to it (Seo et al., 2015).

All these findings corroborate that the emotional bond and attachment that people feel toward robots is comparable to attachment to humans. Given this, for effective robot-based therapy, the relationship between the client and the robot therapist has to have at least the potential to develop the critical features that define an attachment bond. In such a therapeutic relationship, the client: (1) seeks proximity to the robot therapist in the form of an emotional connection that deepens over the course of regular meetings; (2) is willing to rely upon the robot therapist as a safe haven when feeling threatened or psychologically injured; (3) derives a sense of felt security from the robot therapist, who then functions as a secure base to facilitate healthy growth; (4) experiences separation anxiety when the robot therapist is temporarily unavailable, or as the anticipated end of the relationship approaches; and (5) perceives the robot therapist as stronger and wiser due to its training and experience.

## The major roles of social robots in mental health settings

Not all roles that robots can play in mental health settings require the same level of attachment that ideally characterises therapeutic relationships. David et al. (2014) categorize the possible roles as follows. As *assistants or tools*, social robots are used for assessment/diagnosis and the development and practice of social skills. As *mediators*, robots enable or facilitate the progress of treatment by acting as an intermediary in interactions between the therapist and the client, and are sources of motivation and encouragement, rendering the treatment engaging. Finally, as *therapists or coaches*, robots themselves deliver psychotherapy (while their activities are determined and overviewed by practitioners).

**Figure 1:**
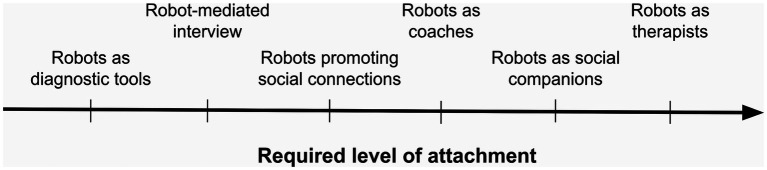
An ordering of the roles robots can play in mental health settings in terms of the level of attachment required by the specific roles in question.

This categorization, however, is too coarse-grained and oversimplifies the distinctions between different use cases. An analysis of the literature reveals at least six different categories. Robots can be used as *diagnostic tools*, *interview mediators*, *promoters of social connections*, *coaches*, *social companions* and *therapists*. As we argue below, the strength of attachment evoked by these different roles is distinct in each case, which allows us to systematically position these roles along the dimension of the required level of attachment (see Figure 1).

### Robots as diagnostic tools

Applied together with traditional interviews and self-report inventories, robots can help behavioral assessments and inform a more thorough diagnostic evaluation. For example, in the context of diagnosing autistic spectrum disorder (ASD), robots can elicit social responses, which then have high diagnostic value (Diehl et al., 2012). Similarly, in the case of social anxiety, robot-based behavioral assessment tests have discriminative validity in distinguishing between young people with and without social anxiety disorder (Rasouli et al., 2022).

Given that this type of connection is temporary and does not aim to develop the feeling of safe haven or secure base functions, for this type of usage only a low level of attachment is needed (if any, as attachment usually does not play a role in one’s relation to, e.g., a paper-and-pencil type of personality test).

### Robot-mediated interview

In case of excessive social anxiety, interview situations and initial treatment sessions may be anxiety-provoking—and this could interfere with the assessment process. One way to decrease these negative effects could be the involvement of a social robot in the interview process. Studies show thatthe responds from children, their engagement and the content provided (i.e., amount and types of information) are similar in the case of robotic interviewers than in the case of a human interviewer (Wood et al., 2013a,b). Some children with special needs are even more interested in and cooperate better with robotic than human interviewers. For instance, when interviewed by a robot, children reported occurrences of bullying significantly more (Bethel et al., 2016).

Since in such cases of robot-mediated interviews, the connection to the robot is established by mutual (verbal and non-verbal) communication, its attachment-evoking effect is theoretically stronger than in the case when a robot plays the role of a purely diagnostic tool.

### Robots promoting social connections

One of the main goals of rehabilitation and therapy processes is to increase the frequency of prosocial behaviors. As certain findings suggest, a robot can encourage such desirable prosocial behavior. A robot can elicit and guide joint attention (Dautenhahn, 2003) and can serve as a “catalyst” for social interactions with another individual (Feil-Seifer and Matarić, 2011). Human social mediators can facilitate human interactions in various social settings, such as school classrooms or formal meetings. In a similar vein, it is possible to use a robot as a social mediator (Gillet et al., 2020). Studies show that social robots acting as mediators have been effective in improving human interaction through numerous aspects, such as facilitating conversation, engagement, task collaboration and participation (Adikari et al., 2023). For example, a robot can encourage a child with ASD to interact with an interlocutor who is present in the therapeutic setting (Dautenhahn, 2003). Similarly, it has been observed that two children with ASD continued playing a ball game with each other after learning it from a robot (Costa et al., 2010).

This kind of connection means more involvement than a “simple” interview but requires less attachment than coaching, which is typically a longer-term relationship with the specific target of changing at least certain behavioral patterns.

### Robots as coaches

Social robots can act as instructors or a coaches to monitor and engage users in a highly personalized way to improve their social, physical, or cognitive well-being. For instance, the social robot Autom is a behavior change coach facilitating sustained engagement in a diet and exercise program (Kidd and Breazeal, 2008). It can track participants’ weight loss and can provide personalized feedback. According to the findings, this form of coaching is more engaging than computerized or paper-based logs, and the participants felt a form of working alliance and close relationship with the robot. Jeong et al. (2023) used Jibo, a social robot companion as a positive psychology coach to provide positive psychology coaching for university students. After seven sessions of interacting with the robot, participants showed statistically significant improvement in their psychological well-being, mood, and readiness to change health-related behaviors. Students expressed appreciation for the robot’s companionship, desire to talk and communicate with it, and the feeling of attachment to it.

As this type of role in general necessitates a longer relationship than the previous one with the added goal of changing behavioral patterns, it also requires stronger attachment. Compared to social companionship (see below), however, it is less demanding, since whereas in coaching the connection between the robot and the client is extrinsically motivated and focuses on some external reinforcement, in social companionship the motivation of the connection is much more intrinsic.

### Robots as social companions

Increased loneliness and social isolation may affect a third of the world’s population, and come with serious health-related consequences, such as increased risk for mental illness, obesity, dementia, and early death (Broadbent et al., 2023). Without a doubt, the most ideal solution for this problem would be a human companion for everyone. But in reality, it is increasingly difficult to make new friends as an adult, so relying on companion robots to support socially isolated adults may prove to be a promising compromise. As investigated in the context of elderly care and children with ASD, the relation to social companion robots seems to be still ambivalent. Konok et al. (2018), for instance, compared people’s reactions to social robots and dogs and found that people’s attitude toward robots is much more negative than toward dogs. The main advantage of dogs over robots seems to be the presence of emotions (e.g., love, faithfulness, kindness) and attachment behavior (e.g., the dog seeks the proximity of the owner and shows stress behavior when separated from the owner).

In the future, newer robots leveraging advanced AI algorithms may foster stronger social connections with humans than earlier generations of robots. Generative AI, like ChatGPT, which is based on large language models, allows robots to engage in more spontaneous conversations, which will support the social companion role.

In addition to the advancement of AI algorithms, the results from studying human-dog connections could also shape human-robot attachment. Based on the differences between the attachment to dogs and social robots, Konok et al. (2018) suggest the following considerations for planning more attachment-prone social robots. The attitudes toward companion robots might be improved by implementing behaviors that trigger the users to attribute emotions and personality to the robot. Based on the fact that in the case of dogs it is preferred if they are not perfectly obedient, minor disobedience and imperfectness might render robots more “real” or “alive” as if they had their own personality. Similarly to dogs, robots or artificial agents should also be equipped with an ‘attachment system’ (Kovács et al., 2011; Ichikawa et al., 2012) that is able to recognize, prefer and maintain proximity to their users, show signs of stress when separated from and greet them happily when reunited with the users (Konok et al., 2018). Based on pet-attachment results, initiating physical contact might also be useful, if the physical parameters of the robot make it possible, safe (see, e.g., Haddadin et al., 2008), enjoyable and if it fits the personality and preferences of the user (Walters et al., 2005).

### Robots as therapists

As already mentioned, one of the main goals of the therapist during any kind of psychotherapy is to act as an attachment figure and create a secure base for the patient to enable exploration of attachment-related experiences and to provide a corrective emotional experience. This corrective emotional experience can reinforce the patient’s capacity for reflective functioning (i.e., to make inferences about the mental states of oneself and others; see Fonagy et al., 1996). Whereas in the case of coaching the focus is on behavioral change, in the case of therapy, the goal often includes improving self-reflection, self-knowledge, and the capacity for mentalization. These goals require a high level of attachment. One could argue that the social companion role requires stronger attachment than the therapist role, as the latter only relies on an “alternative support connection” (Saunders et al., 2011). However, whereas psychotherapy does have indeed an “unrealistic” relationship layer (based on transference and countertransference), it has a real relationship feature as well that is based on attachment and secure rapport (Saeed, 2000).

We can only speak about robot-led psychotherapy if the “robot therapist” can accomplish these requirements for attachment. Various studies show that social robots prompt people to develop emotional bonds (Sharkey and Sharkey, 2021; Law et al., 2022), and it is also known that forming strong emotional bonds often leads to the development of attachment relations (Bowlby, 1982; Cassidy and Shaver, 2016) and that physical availability and emotional connection are the two main communication channels that are relevant in attachment formation (Gagliardi, 2021). However, it is still an open question whether the features of this kind of attachment are suitable for reaching the full potential of psychotherapy.

### The future of human-robot attachment in mental health care

Because of the fundamentally social nature of humans, our level of social connectedness is strongly associated with the markers of mental and physical well-being (Kim and Sul, 2023). In the case of treatments in a mental health context, the process of healing (i.e., the speed of recovery) heavily depends on the quality of the attachment between the client and the patient (Mallinckrodt, 2010).

In this paper, we have shown that it is possible to distinguish between different use cases from this perspective. Some roles that robots could play in mental health do not rely on attachment but others can only be effective if the robot can accomplish the safe haven function.

For future developments in the area, it is crucial to focus on the formation of emotional bonds between robots and patients, ideally from an integrative point of view combining social neuroscience, computer science and robotics (see, e.g., Cross et al., 2019). It could also be fruitful to examine the neurological responses of clients to social robots in different types of helping relationships. We hypothesize that at a lower level of attachment (e.g., robots as diagnostic tools) the neurological responses to the robot will be different than at a higher level (e.g., robots as therapists).

As Kaplan (2001) stated, the “Turing test” for social robots is to “pass” the attachment test; that is, to show and trigger attachment behavior to and from the user. Reaching this level of development, the next question is the effect of human-robot attachment. In “traditional” (that is human-led) therapies the secure attachment between the patient and the therapist has the potential to modify the insecure attachment working model of the patient. In successful therapy, the secure base and safe haven functions of the therapist become *generalized* to other connections. Future studies have to answer whether this generalized positive effect can occur in robot-based psychotherapy as well. Ideally, it should happen because the *final and utmost goal of any kind of psychotherapy is to help the patient build deeper, more satisfying and secure connections with other humans.*

## Conclusion

Social robots could be used in the full spectrum of mental health care, they could decrease the so-called “treatment gap” (the burden implied by the lack of human mental health professionals), and they could also increase the quality of treatments. However, for their effectiveness, their capacity to trigger attachment feelings and behaviors in patients will need to be improved and carefully fine-tuned in line with the requirements of the specific roles the robots would play.

## Data availability statement

The original contributions presented in the study are included in the article/supplementary material, further inquiries can be directed to the corresponding authors.

## Author contributions

MS: Conceptualization, Methodology, Resources, Visualization, Writing – original draft, Writing – review & editing. PF: Conceptualization, Writing – review & editing.
